# Supplementary material for: Rapid and sensitive detection of SARS-CoV-2 antibodies by biolayer interferometry
Source: Sci Rep. 2020 Dec 10;10:21738. doi: 10.1038/s41598-020-78895-x (PMC7730435; doi:10.1038/s41598-020-78895-x)
Supplement: Supplementary file 1 — Supplementary Information. [file 41598_2020_78895_MOESM1_ESM.pdf]

Supplementary Information

Rapid and sensitive detection of SARS-CoV-2 antibodies by biolayer interferometry

John V. Dzimianski[1], Nicholas Lorig-Roach[1], Sara M. O'Rourke[1], David L. Alexander[2], Jacqueline M. Kimmey[3], Rebecca M. DuBois[1*]

[1]*Department of Biomolecular Engineering, University of California Santa Cruz, Santa Cruz, California, USA*

[2]*Ontera Inc., Santa Cruz, California, USA*

[3]*Department of Microbiology and Environmental Toxicology, University of California Santa Cruz, Santa Cruz, California, USA*

[*]*Corresponding author: rmdubois@ucsc.edu*

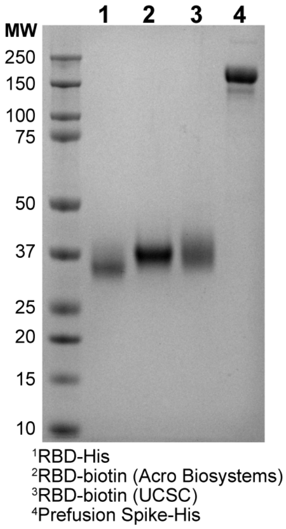

a

Supplementary Figure 1 | Purity of SARS-CoV-2 antigens. Coomassie-stained SDS-polyacrylamide gel of antigen samples used in ELISA and BLI-ISA experiments. Each lane was loaded with 2 μg of sample that had been boiled for 10 minutes in a 1X reducing loading buffer.

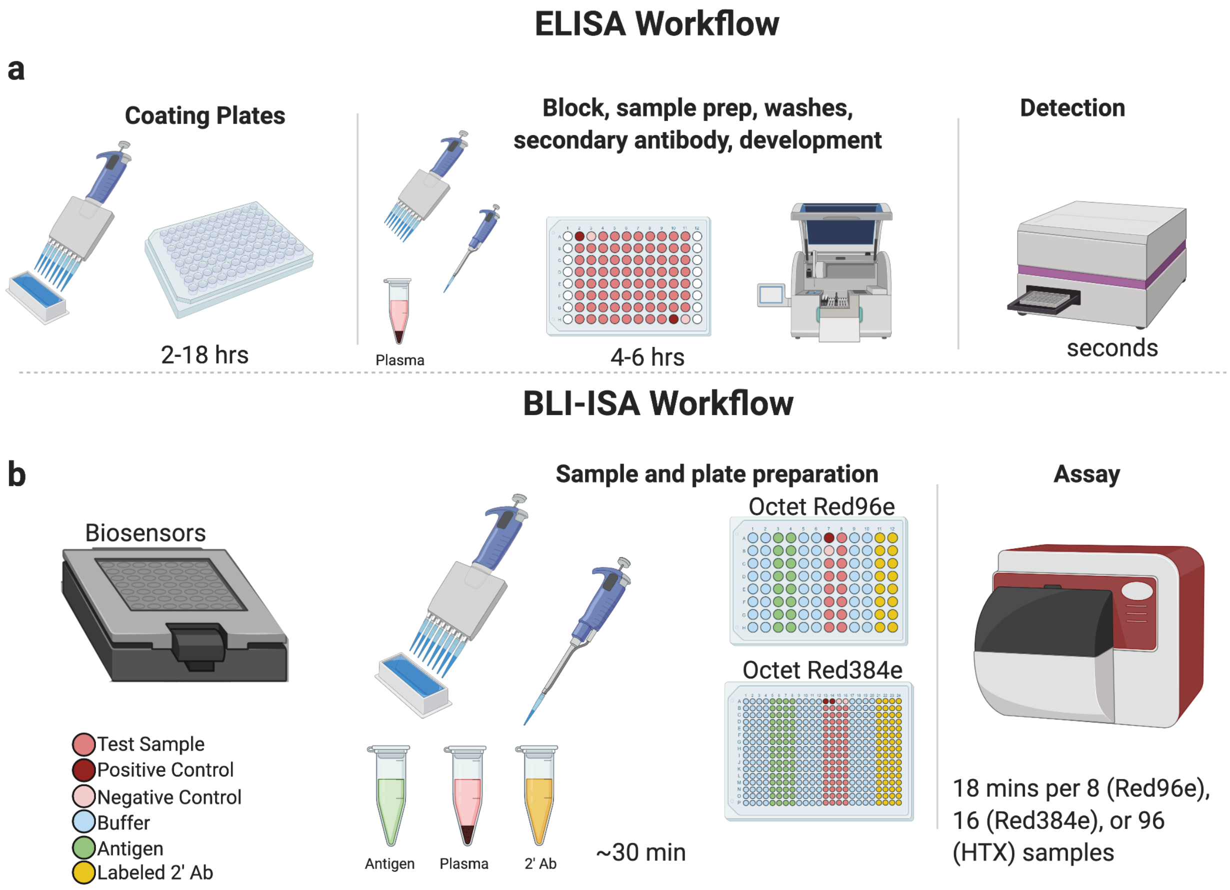

Supplementary Figure 2 | Comparison of labor and assay time for ELISA and BLI-ISA. a In ELISA experiments, preparation of test plates can take hours to overnight for the first step of antigen coating to the plate surface. Following this, there are several hours of labor and incubation steps before detection can be performed. **b** In BLI-ISA experiments, labor time is limited to the preparation of samples and pipetting those samples and buffers into test plates. Created in BioRender.com.

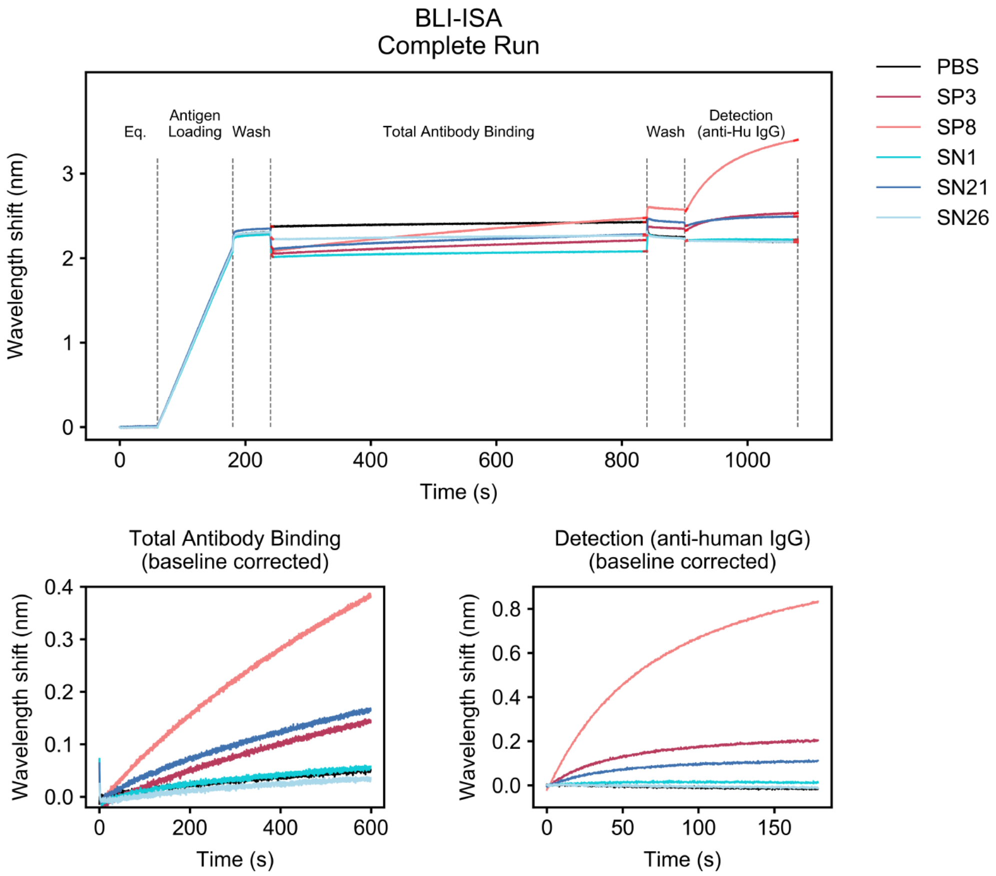

Supplementary Figure 3 | Complete BLI-ISA experiment raw data signals with representative plasma samples and SARS-CoV-2 spike RBD antigen. (Top) Colored traces plot time series signals collected during BLI-ISA experiments. Assay steps (e.g. biosensors dipped into antigen or wash buffers) are denoted with dashed lines and labeled. The initial dip in signal in the first ~2 seconds of the Total Antibody Binding step is different in each plasma sample, and this data is excluded from baseline-adjusted difference measurements (below). Bottom panels show baseline-adjusted differences in signal over time during the Total Antibody Binding (left) and Detection steps (right). The difference in signal between the beginning and end of each association step is reported.

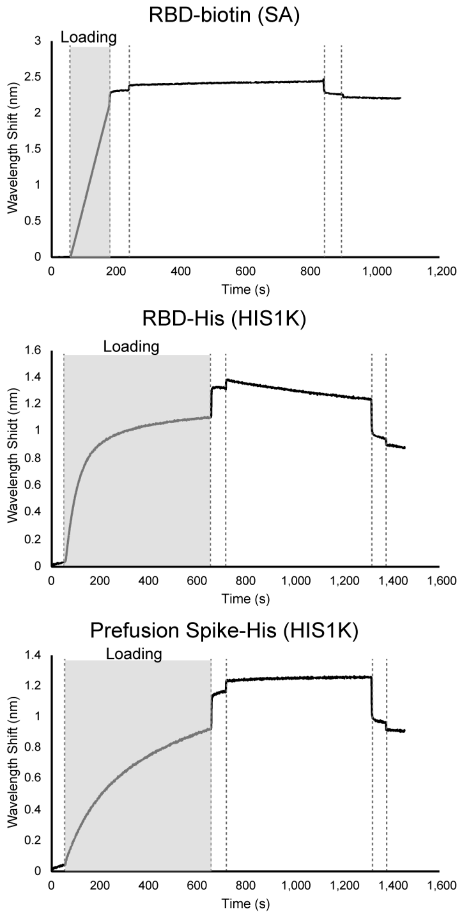

Supplementary Figure 4 | Antigen stability on biosensors. Mock BLI-ISA experiments of different antigens loaded on biosensors and then dipped into assay buffers. (Top) RBD-biotin loaded onto streptavidin SA biosensors has a stable signal over the course of an experiment. (Middle) RBD-His loaded onto anti-penta-His HIS1K biosensors suffers from a baseline drift over the course of an experiment. (Bottom) Prefusion Spike-His loaded onto anti-penta-His HIS1K biosensors has a stable signal over the course of an experiment, presumably due to the stronger anchoring by its three His-tags on the trimeric antigen.

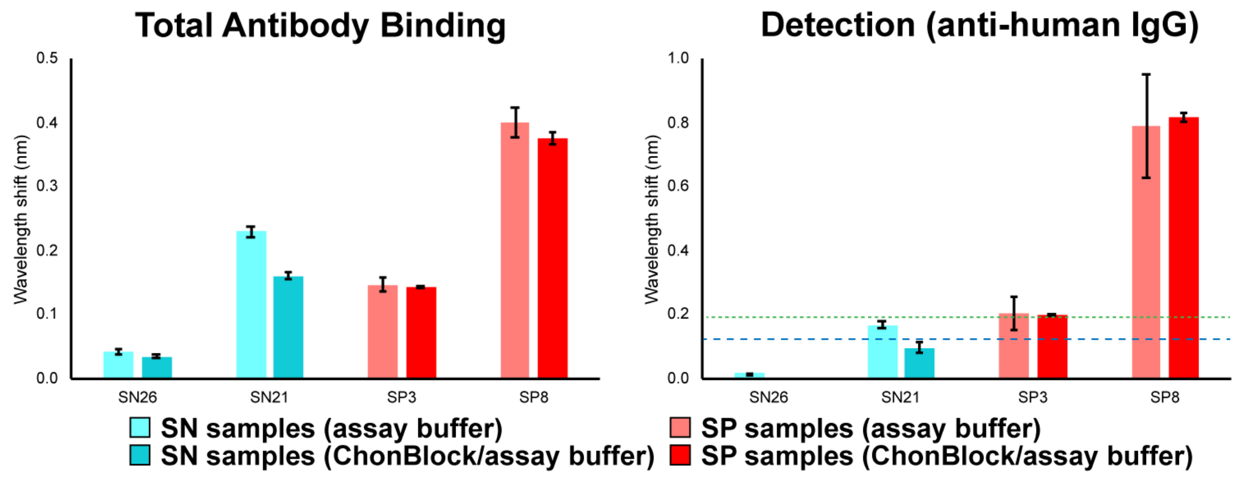

Supplementary Figure 5 | Presence of ChonBlock blocking agent in pre-plasma wash step and plasma dilution buffer reduces background signal. Plasma samples were diluted in BLI assay buffer alone or 20-25% ChonBlock in BLI assay buffer. In both the Total Antibody Binding step (left) and anti-human IgG Detection step (right), presence of ChonBlock reduced the magnitude of signal observed from pre-pandemic seronegative samples (cyan) but not convalescent seropositive samples (red). The assays were performed with plasma at a 1:8 dilution. Bars represent the mean of biological duplicates, and error bars represent one standard deviation from the mean. Blue and green dashed lines represent the mean of all seronegative samples (see Fig. 3) plus 3 and 5 standard deviations, respectively.

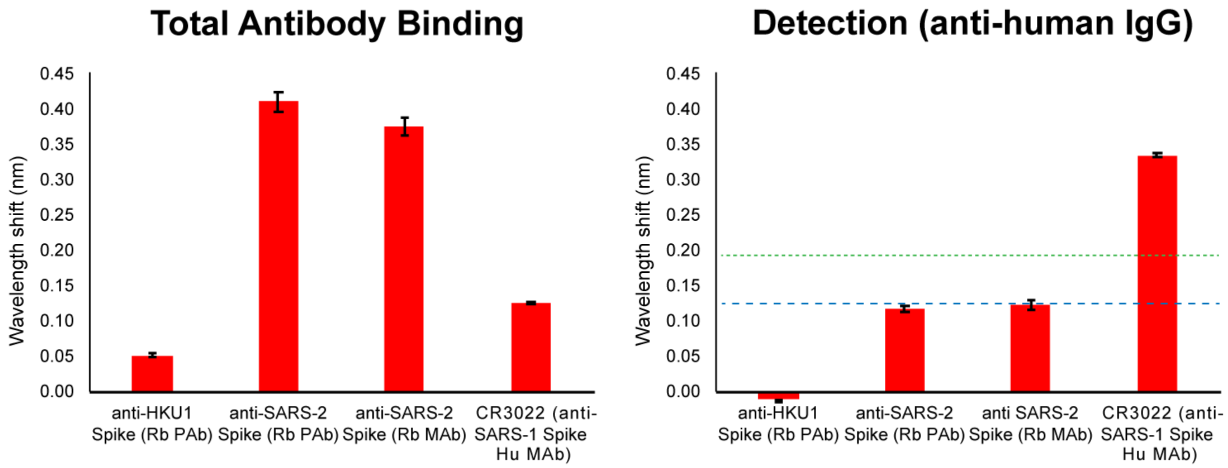

Supplementary Figure 6 | Antigen and anti-human IgG reagent specificity in BLI-ISA. Single-dilution BLI-ISA to evaluate the reactivity of rabbit and human antibodies to SARS-CoV-2 spike RBD. The Total Antibody Binding signal is measured when RBD-biotin-loaded SA biosensors are dipped into four anti-coronavirus spike antibody samples. The Detection signal is measured when RBD-biotin-loaded SA biosensors that had been dipped into the anti-coronavirus spike antibody samples are subsequently dipped into colloidal gold-conjugated anti-human IgG. The rabbit polyclonal antibody (Rb PAb) samples anti-HKU-1 Spike and anti-SARS-CoV-2 Spike were assessed at 1.5 µg/mL. The rabbit monoclonal antibody (Rb MAb) sample anti-SARS-CoV-2 was assessed at at 0.335 µg/mL. The human monoclonal antibody (Hu MAb) sample CR3022, an anti-SARS-1 Spike antibody that cross-reacts with SARS-2 Spike, was assessed at 0.2 µg/mL. Bars represent the mean of biological duplicates, and error bars represent one standard deviation from the mean. Blue and green dashed lines represent the mean of all seronegative samples (see Fig. 3) plus 3 and 5 standard deviations, respectively.

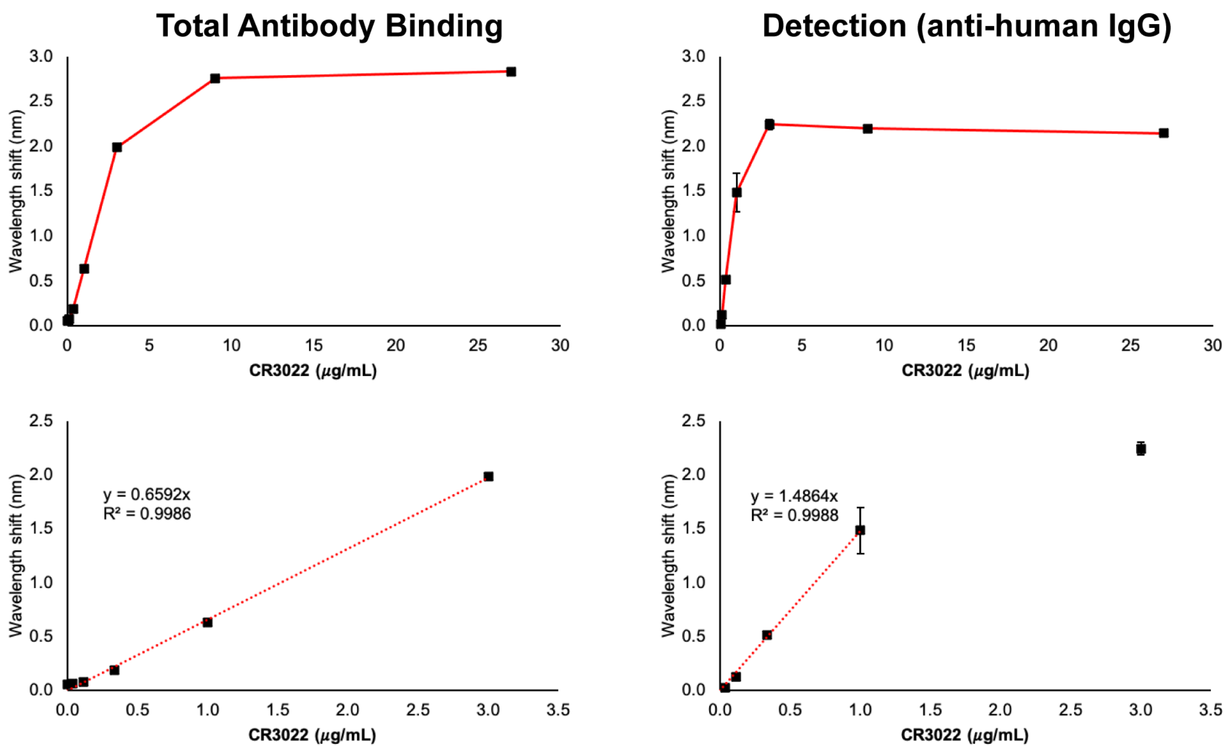

Supplementary Figure 7 | Dose-dependence of monoclonal antibody CR3022 in BLI-ISA. The Total Antibody Binding signal is measured when RBD-biotin-loaded SA biosensors are dipped into a concentration series of monoclonal antibody CR3022 from 0.037-27 µg/mL in BLI assay buffer. The Detection signal is measured when RBD-biotin-loaded SA biosensors that had been dipped into the CR3022 antibody samples are subsequently dipped into colloidal gold-conjugated anti-human IgG. Top panels represent data from the whole concentration series, whereas bottom panels represent data in the linear dose-dependent range. Squares represent the mean of biological duplicates, and error bars represent one standard deviation from the mean.

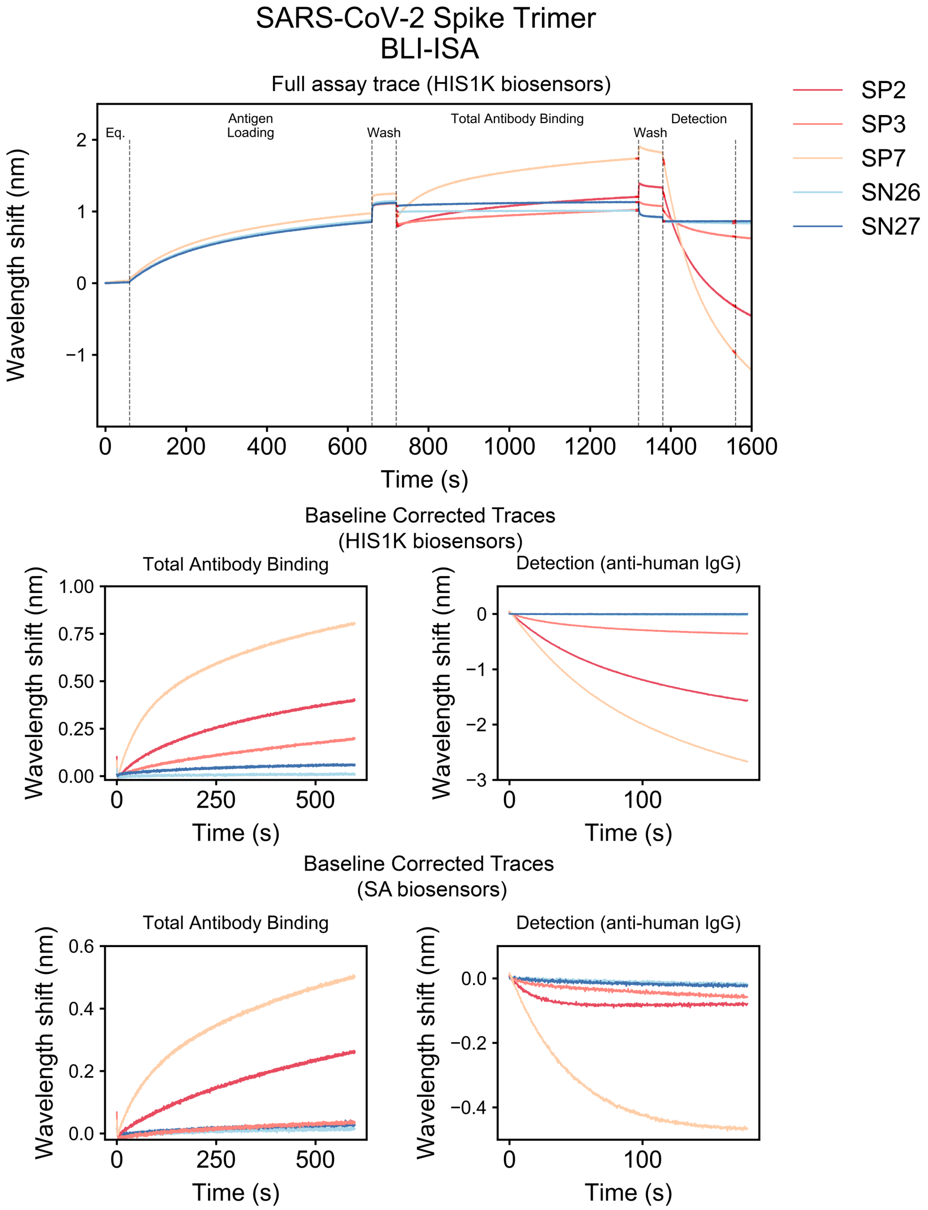

Supplementary Figure 8 | Complete BLI-ISA experiment raw data signals with representative plasma samples and SARS-CoV-2 prefusion Spike antigen.

(Top) Colored traces plot time series signals collected during the BLI-ISA experiments. Assay steps are denoted with dashed lines and labeled. Middle panels show baseline-adjusted differences in wavelength shift over time during the Total Antibody Binding (left) and Detection steps (right) using prefusion Spike immobilized on HIS1K biosensors. Bottom panels show baseline-adjusted differences in wavelength shift over time during the Total Antibody Binding (left) and Detection steps (right) using prefusion Spike immobilized on SA biosensors. Note in the anti-human IgG detection step, there is an inverse shift in wavelength relative to what is observed for RBD antigen (Fig S3).
